# Measuring Empathy among Dental Students and Interns: A Cross-Sectional Study from Dammam, Saudi Arabia

**DOI:** 10.1155/2021/5584423

**Published:** 2021-04-27

**Authors:** Muhammad Nazir, Muhanad Alhareky, Abdulrahman Alqahtani, Leenah Alsulaimi, Reema Alotaibi, Najwa Yousef, Fatimah Abushal, Jehan Alhumaid

**Affiliations:** ^1^Department of Preventive Dental Sciences, College of Dentistry, Imam Abdulrahman Bin Faisal University, P. O. Box 1982, Dammam 31441, UAE; ^2^College of Dentistry, Imam Abdulrahman Bin Faisal University, P. O. Box 1982, Dammam 31441, UAE

## Abstract

**Objective:**

To evaluate empathy and its related factors among undergraduate dental students and interns enrolled in a public dental college in Dammam, Saudi Arabia.

**Materials and Methods:**

This cross-sectional study used the Jefferson Scale of Empathy-Health Profession Students (JSE-HPS) version to determine empathy in 362 dental students and interns in Dammam, Saudi Arabia. The JSE-HPS is a 20-item 7-point Likert scale questionnaire, and its score ranges from 20 to 140 with high values indicating increased empathy. Influences of age, gender, class year, previous year's grade point average (GPA), educational attainment of parents, and monthly family income on empathy were evaluated.

**Results:**

Of 501 enrolled students and interns, 362 returned completed questionnaires, and the response rate of the study was 72%. The sample's empathy score (JSPE-HPS scale) ranged from 70 to 129 with a mean of 96.75 (±13.76). Most participants believed that empathy is important for effective communication with patients (96.1%) and can improve the provider-patient relationship (95.6%). Females demonstrated a significantly higher mean empathy score (99.98 ± 14.01) than males (92.72 ± 12.35) (*P* < 0.001). Similarly, the participants with high GPA (98.06 ± 13.69) had significantly greater mean empathy scores than those with low GPA (94.84 ± 13.68) (*P*=0.029). The mean empathy score increased significantly from junior students (3^rd^ and 4^th^ year students) to senior students (5^th^ and 6^th^ year students) and interns (*P*=0.008). Multiple linear regression analysis showed that class year (*B* = 2.03, *P*=0.006) and GPA (*B* = 8.67, *P*=0.003) were significant factors associated with empathy.

**Conclusions:**

Empathy is important for effective patient communication and improved provider-patient relationship. Female gender, high GPA, and class years were associated with empathy. Empathy should be integrated into dental curricula for effective student learning and positive patient care outcomes.

## 1. Introduction

Empathy, an important phenomenon in patient and healthcare provider relationship, is the ability of an individual to stand in the shoes of another person to better understand his/her perspective or situation [1, 2]. In healthcare, empathy is defined as “a predominantly cognitive attribute that involves an understanding of the patient's experiences, concerns, and perspectives, combined with a capacity to communicate this understanding and an intention to help” [3]. There are studies about an association between empathy among healthcare providers and positive clinical outcomes [3, 4]. It was reported that diabetic patients treated by physicians with higher scores of empathy demonstrated significantly improved control of hemoglobin A1c than those patients who were treated by physicians with low empathy scores [3]. Similarly, diabetic patients who received treatment by physicians with a high empathy score showed significantly lower acute metabolic complications than the patients of physicians with moderate and low levels of empathy [4].

It is well documented that empathy is associated with improved enablement, increased participation and education, higher compliance, reduced emotional distress, and improved patient's quality of life [5–7]. On the other hand, lack of empathy can frustrate and disappoint patients who tend to avoid visiting healthcare providers [8]. In dental literature, empathy is known to reduce dental fear, improve the provider-patient relationship, enhance patient cooperation and compliance, and bring positive clinical outcomes and high patient satisfaction [9, 10]. A study of students in five health sciences professions (dentistry, pharmacy, medicine, veterinary medicine, and nursing) found that the students of dentistry and nursing undergraduate programs had the highest mean empathy scores in the beginning of their first year [11]. There is also evidence about higher levels of empathy in female students compared with male students of dentistry and other healthcare professions [11–13].

Empathy scores among students can be increased and maintained significantly by giving them lectures and showing/discussing videos on empathy for improved patient care [14]. However, there are reports about the decline of empathy among medical and dental students [5, 10, 11]. For example, the first year dental students exhibited significantly higher levels of empathy than students of subsequent years [10]. Similarly, another study reported the highest mean empathy score in the first year (117.23 ± 14.19) and the lowest mean empathy score in postgraduate dental students (108.77 ± 9.12) [15].

There is unquestionable importance of empathy in clinical practice; however, its various aspects have not been fully investigated in dentistry. Therefore, it is important to evaluate the influence of gender, grade point average (GPA), class year, and socioeconomic status factors on the perception of empathy. The understanding of empathy among students and interns can be used to improve the dental curricula and ensure a high quality of patient care. The current study aimed to evaluate empathy and its related factors among undergraduate dental students and interns enrolled in a public dental college in Dammam, Saudi Arabia.

## 2. Materials and Methods

### 2.1. Participants

This cross-sectional study was conducted on dental students and interns at the College of Dentistry, Imam Abdulrahman Bin Faisal University, Dammam. The students spend one year in learning basic sciences and language courses at the preparatory college at the university, and they join their undergraduate dentistry program in second year. That is why the students in the first year were excluded from the study. The second year students were also excluded from the study because they are not exposed to clinical training and patient care. Therefore, the students receiving clinical training and providing patient treatment (3^rd^ year through 6^th^ years at the college) and interns were eligible to participate in the study. The recruitment of participants was carried out by using a census sample of all 3^rd^–6^th^ year dental students and interns. There are 419 students in third, fourth, fifth, and sixth year classes and 82 interns (*N* 419 + 82 = 501). Those students and interns who provided their verbal informed consent received a self-administered paper-based questionnaire.

### 2.2. Study Instrument

The questionnaire can be broadly divided into three sections. The first section of the questionnaire included demographic profiles of participants such as age, gender, class year, previous year grade point average (GPA), educational attainment of parents, and monthly family income. The second section included the Jefferson Scale of Physician Empathy-Health Profession Students (JSPE-HPS) version adapted from Hojat et al., 2001 [16]. There are 20 items in the scale which assess health profession students' self-reported empathy. Half of these items are positively worded statements and the other half are negatively worded statements. A 7-point Likert scale (1 = strongly disagree; 7 = strongly agree) is used for these items. The score of the JSPE-HPS version ranges 20–140. Previous studies performed the psychometric analysis of the JSPE-HPS version and showed that the instrument is valid and reliable to evaluate empathy levels among health profession students [2, 17]. The third section of the questionnaire included six questions about the importance of empathy in improving patient communication, dentist-patient relationship, positive treatment outcomes, quality of dental care, patient satisfaction, and reducing rates of patient litigation. The questionnaire was also reviewed to evaluate its appropriateness from the cultural point of view.

### 2.3. Ethical Considerations

Aims of the study including its details were discussed with study participants. They were informed about their right to voluntary participation in the survey. The privacy and confidentiality of their responses were ensured by an anonymous survey. Explanations about the items of the questionnaire were provided to the study participants. Self-administered paper-based questionnaires were administered in classes after students finish their lectures. Dental interns received the questionnaire in their clinics in the dental hospital. Two subsequent visits were made if interns were unable to provide their responses in the first visit due to their clinical commitments. The scientific research unit (ethics committee) at the college provided ethical approval of the study (EA 202016) on 10/11/2019. Ethical standards of the Helsinki Declaration were followed during the study.

### 2.4. Statistical Analysis

Data were entered in MS Excel and then transported and stored in the SPSS version 22 (IBM, Armonk, NY, USA). Descriptive statistics included means, standard deviations, frequencies, and proportions. The first 10 negatively worded items were reverse coded; then, the total empathy score for each participant was calculated by adding the responses to 20 items of the JSPE-HPS scale, and finally, the mean empathy score of the sample was calculated. Analytical statistics were performed using the independent-sample Student's *t*-test and one-way ANOVA test. These tests compared mean empathy scores between male and female students and among students from different years of study, parental education, and monthly family income (low income ≤ 5000 SAR/month, middle income 5001–20000 SAR/month, and high income > 20000 SAR/month). One thousand SAR is equivalent to $US266. The bivariate analysis included using the Pearson's correlation test. Multiple linear regression analysis was also performed to evaluate the factors that independently correlated with the empathy score. A *P* value <0.05 was considered statistically significant.

## 3. Results

Three hundred and sixty-two students and interns participated in the study with 44.5% of males and 55.5% of females. The response rate was 362/501 = 72%. Most participants (59.4%) had high GPA (4-4.9) and had college/university educated fathers (65.5%) and mothers (55.2%). Three-quarters of the participants reported that empathy was taught in their undergraduate course. The empathy score (JSPE-HPS scale) of the sample ranged from 70 to 129 with a mean of 96.75 (±13.76). Females demonstrated a significantly higher mean empathy score (99.98 ± 14.01) than males (92.72 ± 12.35) (*P* < 0.001). The mean empathy score increased significantly from junior students (3^rd^ and 4^th^ year students) to senior students (5^th^ and 6^th^ year students) and interns (*P*=0.008). The mean empathy score was significantly higher in participants with high GPA (98.06 ± 13.69) than those with a low GPA (94.84 ± 13.68) (*P*=0.029). The empathy score rose with increasing parental educational levels and monthly family incomes; however, differences in mean scores were not statistically significant. The participants who said empathy was taught in the course had a higher mean empathy score (97.15 ± 14.09) than those who denied learning empathy (95.6 ± 12.73) (*P*=0.351) (Table 1).

Nearly 96.1% of the participants believed that empathy is important for effective communication with patients, and 95.6% believed that having empathy can improve the provider-patient relationship. The participants who believed in the role of empathy in effective communication, improved provider-patient relationship, positive treatment outcomes, enhanced quality of patient care, and improved patient satisfaction that demonstrated significantly higher mean empathy scores than those who did not believe in these characteristics (Table 2).

Bivariate linear regression analyses showed that class year significantly correlated with the empathy score (*r* = 0.15, *P*=0.017). Similarly, there was a significant correlation between GPA and the empathy score (*r* = 0.22, *P*=0.001) (Table 3).

In the multiple linear regression model, class year (*B* 2.03, *P*=0.006) and GPA (*B* 8.67, *P*=0.003) remained significant factors associated with empathy among study participants. As the class year increases by one unit, empathy among study participants increases by a factor of 2.03. Similarly, a unit increase in GPA increases empathy by a factor of 8.67. On the other hand, parental education and monthly family income showed no significant relationship with empathy (Table 4).

## 4. Discussion

The study aimed to assess levels of empathy and its influencing factors among dental students and interns. It was found that the female gender, high GPA, and senior class years/internship were significantly related to high empathy in our sample. The mean empathy score in our study was 96.75 ± 13.76 which is higher than that reported in a recent study of Saudi dental students by Naguib et al. in Jeddah who showed that the mean empathy score was 84.84 ± 11.28 [18]. Dental literature shows varying levels of empathy in dental students in different parts of the world. Based on the Jefferson Scale of Empathy‐Health Profession Students, previous studies reported high mean empathy scores in American dental students (117.71 ± 14.06) [10], Nigerian dental students (104.01 ± 19.64) [19], and Pakistani dental students (101.15 ± 13.73) [20]. Conversely, low mean empathy scores (78–90) were reported in Malaysian, Indian, Iranian, Polish, and British dental students [21–25]. These variations in empathy levels in dental students in different countries may be attributed to the differences in cultures, traditions, religion, emotional status, psychological characteristics, and prosocial behaviors [19, 26].

The researchers from three universities in the U.S. collected data on empathy among 104,365 adults from 63 countries and revealed that Saudi Arabia was the second most empathetic country, whereas the U.S. was ranked the seventh most empathetic country in the world. The authors reported that countries with higher empathy had higher conscientiousness, collectivism, sociability, self-esteem, well-being, emotionality, and prosocial behavior such as volunteerism and help [26]. Despite, Saudi Arabia was ranked among the countries with the highest empathy score globally; however, the empathy score in Saudi dental students was lower than reported in dental students of some other countries [10, 19, 20]. The differences in study design and the measurement tool for the evaluation of empathy may account for inconsistencies in empathy levels in adult populations and dental students.

Globally, women exhibit higher total empathy, empathetic concern, and perspective-taking than men [26]. Literature shows that Malaysian and Indian male dental students demonstrated higher mean empathy scores than female students [23, 24]. However, consistent with previous research in dental students [10, 15, 18, 20], females had significantly higher empathy scores than males in the current study. Similarly, in the majority of studies on medical and other health profession students, the higher empathy score was reported in females than males [2, 12, 13, 27]. The caring nature and emotionally sensitive characteristics of females might be the reason for more empathetic behavior in females than their male counterparts [20, 28]. Factors such as social learning, genetic prediction, and the evolutionary process may also be responsible for gender differences regarding empathy [28].

Our study showed a significant increase in empathy scores from the third year to sixth year and internship. A recent study of Saudi dental students reported higher empathy scores in fifth- and sixth-year students than third- and fourth-year students; however, the difference was not significant [18]. Similar trends were observed among Polish dental students who showed increased empathy scores from the first/second year to the fifth year but with no significant difference [25]. Conversely, previous studies showed significantly higher empathy scores in first-year students than students in the subsequent year of the dentistry program [10, 15]. The increase in the empathy score from early years to later years of the undergraduate program is possibly related to increased exposure to clinical training and greater interaction with patients and clinical practice [18, 25].

In a descriptive cohort study, Carr et al. demonstrated significant correlations between the GPA of students and their workplace performance as junior doctors reflected both in clinical management and communication subscales [29]. It is also known that empathy is associated with clinical competence and communication [27, 30]. Therefore, it was assumed that those participants who have high GPA would be more empathetic than those with low GPA. Our findings confirmed this, and the empathy score was significantly higher in participants with a high GPA than those with a low GPA. In addition, GPA was a significant predictor of empathy after controlling for class year, parental education levels, and monthly family income. However, these findings contrast with the literature on empathy among medical students where Hojat et al. found no significant relationship between empathy and performance in the Medical College Admission Test (MCAT) and the US Medical Licensing Examinations (USMLE) [27].

The development of empathy behavior is important for dental students and the dental profession because of improved clinical outcomes associated with high levels of empathy [3, 28]. In the studies of medical students, the empathy score in students was significantly associated with faculty's rating of clinical competence and communication scores of students [27, 30]. Hence, there is a greater emphasis on empathy among dental students which is evident from the recent surge in the literature on dental education [18–20, 25, 31]. In our study, a vast majority of participants believed in the importance of empathy in effective communication with patients and improved provider-patient relationship. Similarly, those who believed that empathy is important for effective communication and improved provider-patient relationship exhibited significantly higher empathy scores than those who did not believe in the role of empathy in patient communication and provider-patient relationship.

Parental socioeconomic influences are known to affect learning performance and academic growth which can lead to clinical performance in students [29, 32]. The current study investigated the influence of parental education and monthly family income levels on empathy among participants. It was hypothesized that participants with low socioeconomic status would demonstrate low empathy. Our findings confirmed that the lower empathy score was obtained from the participants with low parental education and low family income than those with high parental education and income levels, although differences in the mean scores of empathy were not statistically significant.

The use of a census sample of participants in the current study provided reliable evidence on empathy in dental education. However, there are certain limitations to the study. The evaluation of empathy through self-reported data may not truly reflect the level of empathy that participants may actually demonstrate in dental practice. The data collected from one dental college can limit the external validity of the study. In addition, the cross-sectional design is limited in establishing cause and effect relationships. For instance, it is not possible to ascertain if increased GPA can result in an increased empathy score or increased empathy can lead to a high GPA. Given the important role of empathy in clinical practice, the current study is expected to stimulate further research among students, faculty, and dental practitioners. In the future, a prospective multicenter study design should be used to assess empathy and its relationship with different variables among faculty and students.

## 5. Conclusions

A vast majority of participants believed in the importance of empathy in effective communication with patients and improving the provider-patient relationship. The study showed significantly increased empathy in females than males. Similarly, the participants with high GPA were significantly more empathetic than the participants with low GPA. Additionally, GPA independently and significantly predicted empathy levels. Junior students demonstrated lower empathy which increased with advancing class years. However, socioeconomic status variables showed no significant influence on the empathy score. Dental academia may use the study findings when designing undergraduate curricula to enrich student learning and improve patient care. The results of the study may also have implications for the selection/admission criteria for prospective dental students.

## Figures and Tables

**Table 1:** Association between various sociodemographic factors and empathy among participants.

Variables	*N* (%), *N* = 362	Mean empathy score	SD	95% confidence interval		*P* value
				Lower	Upper	
Gender						
Male	161 (44.5)	92.72	12.35	90.82	94.67	<0.001
Female	201 (55.5)	99.98	14.01	97.93	101.76	
Class year						
3rd year	87 (24)	94.73	14.71	91.6	97.87	0.008
4th year	84 (23.2)	93.24	13.54	90.3	96.18	
5th year	78 (21.5)	99.79	13.98	96.64	102.95	
6th year	86 (23.8)	98.91	12.06	96.32	101.49	
Interns	27 (7.5)	98.55	13.16	93.35	103.76	
Grade point average						
High (4-4.9)	215 (59.4)	98.06	13.69	96.12	99.85	0.029
Low (3-3.9)	147 (40.6)	94.84	13.68	92.64	97.11	
Mother's education						
No formal education	24 (6.6)	92.17	13.47	86.48	97.85	0.198
School education	138 (38.1)	97.64	14.14	95.26	100.02	
College/university education	200 (55.2)	96.69	13.48	94.81	98.57	
Father's education						
No formal education	10 (2.8)	89.9	10.79	82.18	97.62	0.271
School education	115 (31.8)	97.22	14.1	94.61	99.82	
College/university education	237 (65.5)	96.82	13.67	95.07	98.57	
Monthly family income						
Low income	14 (3.9)	90.93	10.69	84.75	97.1	0.296
Middle income	138 (38.1)	97.62	13.69	95.32	99.93	
High income	128 (35.4)	97.16	14.03	94.71	99.62	
I do not know	82 (22.7)	95.65	13.82	92.61	98.68	
Empathy taught in the course						
Yes	270 (74.6)	97.15	14.09	95.51	98.84	0.351
No	92 (25.4)	95.6	12.73	93.11	98.11	

**Table 2:** Association between patient care factors and empathy among participants.

Variables	*N* (%)	Mean empathy score	SD	95% confidence interval		*P* value
				Lower	Upper	
(1) Do you believe that empathy is important for effective communication with patients?	Yes: 348 (96.1)	97.24	13.69	95.77	98.7	<0.001
	No: 14 (3.9)	83.64	7.33	79.77	87.89	
(2) Do you believe that empathy can help lower the rates of patient litigation?	Yes: 321 (88.7)	96.98	14.03	95.44	98.57	0.387
	No: 41 (11.3)	95	11.39	91.54	98.64	
(3) Do you believe that having empathy can improve healthcare provider and patient relationship?	Yes: 346 (95.6)	97.22	13.69	95.79	98.82	0.003
	No: 16 (4.4)	86.75	11.48	81.6	93.1	
(4) Do you believe that empathy can lead to positive treatment outcomes?	Yes: 337 (93.1)	97.65	13.69	96.13	99.1	<0.001
	No: 25 (6.9)	84.6	7.54	81.83	87.68	
(5) Do you believe that empathy can improve the quality of patient dental care?	Yes: 332 (91.7)	97.21	13.95	95.63	98.73	0.034
	No: 30 (8.3)	91.67	10.36	88.18	95.36	
(6) Do you believe that empathy is important for achieving patient satisfaction?	Yes: 334 (92.3)	97.24	13.9	95.72	98.74	0.02
	No: 28 (7.7)	90.96	10.7	87.03	94.92	

**Table 3:** Bivariate linear regression analyses: correlation between sociodemographic factors and empathy score in study participants.

Correlation	Pearson's correlation coefficient (95% CI)	*P* value
Class year and empathy score	0.15 (0.04, 0.27)	0.017
GPA and empathy score	0.22 (0.01, 0.34)	0.001
Mother's education and empathy score	0.05 (−0.08, 0.18)	0.459
Father's education and empathy score	−0.02 (−0.15, 0.11)	0.810
Monthly family income and empathy score	−0.01 (−0.14, 0.12)	0.910

**Table 4:** Multivariable linear regression analyses: correlation between sociodemographic factors and empathy among study participants.

Independent variables	Coefficients				*P* value
	Unstandardized coefficients		Standardized coefficients		
	*B* (95% confidence interval)	SE	Β coefficient	*t*	
Class year	2.03 (0.57, 3.47)	0.74	0.18	2.76	0.006
GPA	8.67 (2.97, 14.41)	2.89	0.19	2.99	0.003
Mother's education	0.803 (−2.179, 3.800)	1.517	0.036	0.529	0.597
Father's education	−0.681 (−4.005, 2.625)	1.683	−0.027	−0.405	0.686
Monthly family income	−0.661 (−4.005, 2.625)	1.111	−0.040	−0.595	0.552

## Data Availability

The SPSS data file used to support the findings of this study is available from the corresponding author upon request.
